# Perceived acceptability of and willingness to use syringe vending machines: results of a cross-sectional survey of out-of-service people who inject drugs in Tbilisi, Georgia

**DOI:** 10.1186/s12954-019-0292-8

**Published:** 2019-03-21

**Authors:** David Otiashvili, Irma Kirtadze, Irina Vardanashvili, Mzia Tabatadze, Allison J. Ober

**Affiliations:** 1 Addiction Research Centre Alternative Georgia, 14A Nutsubidze Street, Office 2, 0177 Tbilisi, Georgia; 2 School of Business, Ilia State University, 3/5 Kakutsa Cholokashvili Ave., 0162 Tbilisi, Georgia; 3 School of Arts and Sciences, Ilia State University, 3/5 Kakutsa Cholokashvili Ave., 0162 Tbilisi, Georgia; 4 RAND Corporation, 1776 Main Street, PO Box 2138, Santa Monica, CA 90407-2138 USA

**Keywords:** Injection drug use, HIV, Syringe vending machine, Tbilisi

## Abstract

**Background:**

The growing HIV epidemic in Eastern Europe and Central Asia has been driven by high rates of injection drug use. The Republic of Georgia has among the highest injection drug use rates globally, with a prevalence of 2.24%. The reach of evidence-based HIV prevention interventions like needle and syringe programs (NSP) among people who inject drugs (PWID) has remained below rates that could significantly impact the epidemic. Syringe vending machines (SVM) are an effective and cost-effective supplement to standard NSP; if acceptable to PWID, SVM could reach hard-to-reach PWID and cover geographic areas where fixed or mobile NSPs do not operate. The aim of this study was to assess the perceived acceptability of SVM among out-of-service (harm reduction or substance use treatment) PWID in Tbilisi, Georgia.

**Methodology:**

Participants were recruited using respondent-driven sampling (RDS) to participate in cross-sectional, face-to-face interviews. We conducted individual interviews using a structured questionnaire that covered participants’ socio-demographics, drug use practices, and perceived acceptability of SVM. Uni-variate analyses were employed for data analysis.

**Results:**

The final sample (*n* = 149) was almost exclusively male with a mean age of 42.2 years and mean years of injection drug use of 14.4 years. Heroin, buprenorphine, and stimulants were the main drugs injected. Eighty-five percent of the sample had never received any harm reduction services, and 30% had never been tested for HIV. Fifteen percent of the sample reported sharing injection equipment with others during last month. All but one participant agreed that PWID would benefit from SVM and 145 (97%) said they would personally use SVM. Ninety percent of those sampled stated that they would use HIV self-tests if available from vending machines. The most highly endorsed features of SVM were provision of free injection equipment, no need to deal with pharmacies, uninterrupted 24/7 access, and availability of HIV self-testing kits.

**Discussion:**

Perceived acceptability of syringe vending machines was extremely high among PWID not currently receiving any harm reduction or treatment services, with strong support indicated for uninterrupted free access to sterile injection equipment, privacy, and anonymity. Introducing SVM in Georgia holds the potential to deliver significant public health benefits by attracting hard-to-reach PWID, reducing unsafe injection behavior, and contributing to HIV testing uptake and linkage to care.

## Background

Population prevalence rates of injection drug use in Eastern Europe and Central Asia (EECA) are extremely high compared with the global rate; rates are 1–2% in Ukraine, 1.6% in Kazakhstan, and > 2% in Russia, compared with a global rate of 0.27% [1, 2]. With an estimated 52,000 people who inject drugs (PWID) [3], the prevalence of injection drug use in Georgia (2.24%) ranks third highest in the world [2]. PWID are a driving force behind the growing human immunodeficiency virus (HIV) epidemic in this region, with an estimated 1.7 million people living with HIV [4, 5]. Since 2010, the annual incidence of HIV has increased in the EECA by 60% [6]. Although HIV prevalence among PWID in Georgia is relatively low (1–4%) [7] at present, the public health consequences of injection drug use are high and include high rates of hepatitis C virus (HCV) (61–92%) [8], as well as multiple social and health problems [7, 9]. About 45% of cumulative HIV cases in the country are attributed to injection drug use [10]. The annual incidence of HIV in Georgia has steadily increased over the last decade by 10–25% [11] and has reached 17.9 per 100,000 population in 2015 [12].

Needle and syringe programs (NSP) are an effective, evidence-based intervention for reducing HIV/HCV transmission via unsafe injection among PWID [13–17]. However, the major problem with NSP implementation in the region is low coverage. To date, no countries have achieved recommended coverage targets for HIV prevention among PWID (60% for NSP and 40% for OST) [18, 19]. In Georgia, implementation of harm reduction programs, including NSP, started in 2003 and since then has expanded in scope and the scale [8]. In 2017, there were 14 fixed sites and 6 mobile harm reduction units operational in the country [20]. In the Georgian context, “harm reduction” refers to low-threshold services that include provision of needles and syringes, condoms, naloxone for overdose prevention, voluntary counseling and testing (VCT) for blood-borne infections, case management and social support, referral to specialized medical and non-medical services, and provision of information and education materials. In 2016, these programs provided HIV/HCV rapid testing to more than 26,000 PWID and their partners [21]. However, only 15–30% of PWID received sterile injection equipment from these programs during the last 12 months [7], and the number of syringes distributed per PWID per year reached 72 in 2015, only a fraction of the internationally recommended target of 200 syringes per PWID per year [18]. About one third of PWID (18,000) in the country are using injection opioids and are at increased risk of overdose [22]. Due to flaws in proper documentation of drug overdose-related cases, official data on opioid-related overdose mortality is virtually nonexistent [21]. An indirect source of related information—a client survey implemented by the Georgian Harm Reduction Network (GHRN)—suggests that there were at least 792 non-fatal and 50 fatal opioid overdoses in the country in 2015 [8]. However, these results also have limitations—double counting of overdose cases cannot be excluded. Although naloxone remains on the list of prescription medications, the Georgian government has agreed to allow distribution to high-risk groups via harm reduction services. In 2016, the GHRN distributed 10,876 ampules of naloxone through its network of low-threshold services [23].

Many countries in the EECA region, including Georgia, are in the process of transitioning from funding provided by the Global Fund to Fight AIDS, Tuberculosis and Malaria (GFATM) to national funding. This transition poses significant challenges to sustainability of harm reduction programs, particularly NSP. Currently NSP in Georgia are fully funded by the Global Fund [8]. In countries from which GFATM has already withdrawn (fully or partially), programs focusing on HIV prevention among PWID were the first to be affected [19, 24, 25]. There is a critical need to adopt innovative approaches to HIV/HCV prevention in order to optimize resource allocation and sustain programs currently receiving external donor support.

Syringe vending machines (SVM) are an effective and cost-effective supplement to standard NSP and can reach hard-to-reach groups and cover geographic areas where fixed or mobile NSPs do not operate [26]. SVMs supply sterile needles and syringes and other injection paraphernalia, condoms, risk reduction and other health information, and contact information for available services, and other minor health supplies. Although evidence on effectiveness is limited, available evidence shows that in addition to providing 24/7 access for regular NSP service users, SVMs are successful in reaching sub-groups that for a variety of reasons do not normally attend fixed site NSPs, such as younger PWIDs and women [26–30]. Non-stigmatized access to sterile equipment, anonymity, and privacy has been reported as important advantages of SVM [31, 32]. In addition, SVMs were shown to be a cost-effective mode of NSP [27]. While there have been some studies examining acceptability of SVM, no studies to date have explored attitudes towards SVM among out-of-service PWID either in the Republic of Georgia, or in EECA—a region greatly in need of supplement harm reduction services.

The aim of the current study was to explore the perceived acceptability of syringe vending machines among out-of-service PWID in Tbilisi, Georgia’s largest city, and to assess barriers to introducing SVMs in local settings. We also aimed to learn about PWID interest and need for a variety of additional items (e.g., sterile equipment, health products, and others) to be potentially distributed via SVMs in the country. This cross-sectional study was part of a formative phase of a parent hybrid (implementation + efficacy) cluster randomized trial—*Georgia Syringe Vending Machine Trial* (*GSVMT*)*—*to evaluate process and effectiveness outcomes of introducing syringe vending machines in Tbilisi.

## Methodology

### Sampling and recruitment

To recruit respondents for this cross-sectional survey, we utilized respondent-driven sampling (RDS), an efficient, peer-driven approach for sampling hard-to-reach populations, including PWID [33–37]. Initial “seeds”—outgoing individuals who are selected to start the recruitment process—were identified through personal contacts of a research staff (principal investigator and co-investigator) that had extensive experience with different groups of PWID in Tbilisi. Seven out-of-service PWID (one female) were non-randomly selected as initial seeds, were instructed regarding the eligibility criteria, and were asked to recruit peers who also inject drugs into the study. Each seed/recruiter was given three unique coupons to hand out to potential participants. Each recruited participant who was confirmed for eligibility and participated in interview was offered to recruit his/her peers and received three recruitment coupons. Coupon IDs linked recruits to recruiters; recruiters were paid 15 Georgian Lari (GEL) for each eligible recruit who participated in the survey (exchange rate 1 USD = 2.45 GEL).

### Eligibility criteria

Eligibility criteria included being 18 years of age or older, residing in Tbilisi for the last 6 months, not using NSP services for at least the past 6 months, not currently enrolled in opiate substitution treatment (OST), reporting injection drug use during the last 30 days verified with venipuncture stigmata, fluency in Georgian, and willingness to participate.

### Data collection

Data collection took place at a harm reduction site in the Saburtalo district of the capital city Tbilisi in July 2018. A research assistant screened for eligibility all potential participants who showed up at the research site (harm reduction facility) with RDS coupons. Those deemed ineligible were thanked for their time and were offered standard services provided by the facility. Data on socio-demographic or other characteristics were not collected from ineligible individuals. Following the screening and consenting procedures, three interviewers (research assistants) administered face-to-face interviews to all eligible participants in a secluded area of the harm reduction site. Prior to conducting the interview, interviewers thoroughly explained the concept of SVM and specifics of its functioning.

### Instrument

The questionnaire consisted of 30 locally developed, close-ended questions, the majority of which were multiple-choice, and covered topics related to socio-demographic characteristics of participants, drug use experience and practices, injection and sexual risk behavior, perceptions and attitudes towards SVM, perceived benefits and disadvantages, willingness to use SVM services, potential locations for installation, and other details related to the operationalization of vending machines. We also offered a predefined list of consumables that could be potentially vended from the machines, and participants had an option to select from the list and to add other items based on their preferences and needs. Interviews lasted 30 min on average. Interviewers conducted interviews on tablet PCs. We used the SurveyCTO® platform (https://www.surveycto.com/) to program the questionnaire on PCs and to allow for immediate transfer of data to a secure, web-based server. All respondents provided written consent to participate in the study. Participants were provided with monetary incentives (20 GEL for participating in interview and 15 GEL for recruiting an eligible participant) to compensate them for their time and efforts.

### Ethics approval

The Bio-ethics Committee of the School of Arts and Sciences, Ilia State University, approved all study procedures. All individuals that came to the research site (both eligible and ineligible) were provided with information regarding available services and were offered rapid HIV and HCV screening and counseling. HIV and HCV screening was performed with rapid antibody tests. Individuals with positive test results were offered linkage to facility for confirmatory testing and inclusion in relevant treatment.

### Analysis

We exported data from the online platform into excel and SPSS v. 20.0. We examined central tendencies (mean, median) and frequencies with measures of variability with 95% confidence level. Network recruitment patterns were analyzed using the network visualization program NetDraw 2.158.

RDS data were analyzed using specially designed RDS analysis tool RDSAT 7.1. Since RDSAT requires network data in order to produce weighted estimates, we determined the size of individuals’ networks with respect to people who inject drugs. For this purpose, we asked participants the following questions: How many drug-using men/women who live in Tbilisi do you know? How many of them do you know personally—you know their names and they know your name? How many of them are 18 and older? How many of them did you see during past month? In addition, we used the result of a recent study that estimated the number of PWID in Tbilisi—22,875 [38].

## Results

### RDS sample

**Fig. 1:**
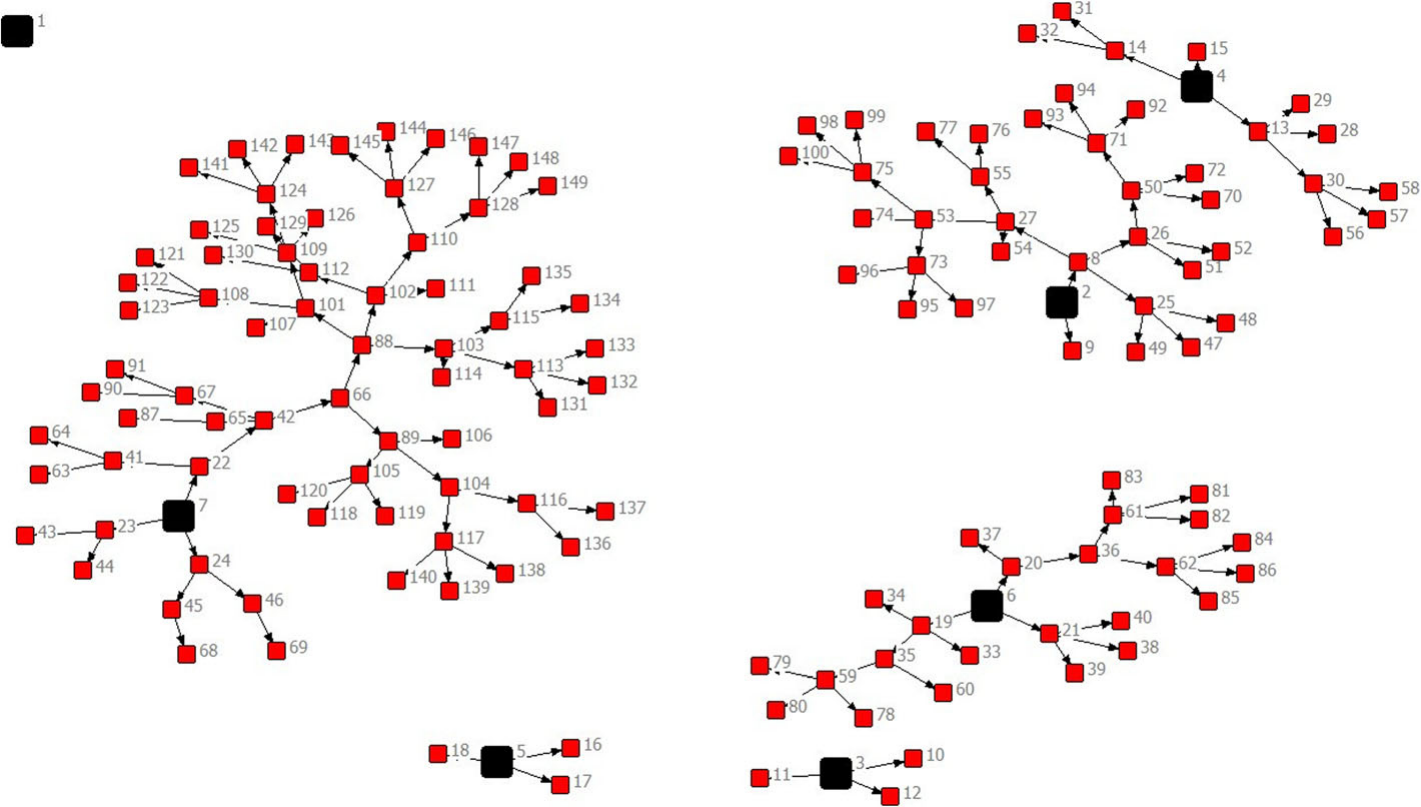
RDS recruitment tree. Figure was produced using network visualization program NetDraw. Black squares represent initial seeds, numbers represent the participant ID number, and arrows show the direction of recruitment. Seed #1 did not recruit any participant, seeds #3 and 5 initiated 1 recruitment wave with 3 eligible participants each, seed #4 initiated 3 waves and recruited 11 participants, seed #6 initiated 4 recruitment waves with 24 eligible participants, seed #2 initiated 5 waves with 31 participants, and seed #7 initiated 8 waves with 70 eligible participants

Figure 1 represents the schematic description of RDS recruitment chains, including the number of waves and eligible participants recruited by each initial seed. Out of 7 initial seeds, 6 were able to recruit eligible participants and 1 (female) failed to do so, as indicated by the single square with 0 connections in the left of the diagram. The minimum number of recruitment waves resulting from a seed was one, resulting in 3 eligible participants; the maximum was eight waves, resulting in 70 participants.

### Socio-demographic characteristics and drug use practices

Out of 172 coupon holders that came to the research site and were screened for eligibility, 149 were confirmed to be eligible and were interviewed and included in the final sample and data analysis. Of 23 individuals not included in the final sample, 11 were excluded because they did not report injection drug use during the last month, 1 was not resident of Tbilisi, 8 were in NSP or OST program at the time of data collection, and 3 were excluded for other reasons. Details of participants’ (*n* = 149) socio-demographic characteristics, drug use and related practices, and history of testing for HIV/HCV are presented in Table 1. The final sample was almost exclusively male (1 female), with mean age of 42.2 (SD 10.9) and a mean history of injection drug use of 14.5 (SD 9.9) years. Forty-five percent had higher education (bachelor or higher) and the majority (73%) was unemployed.

**Table 1:** Participants’ socio-demographic characteristics, injection practices, and history of testing and utilization of prevention services (*N* = 149)

Variable	*N* (%)	95% CI		Mean (SD)	Median (min–max)
		Lower	Upper		
Age		40.45	43.97	42.2 (10.9)	42 (20–63)
Gender					
Male	148 (99)	95.3%	99.9%		
Female	1 (1)	.1%	4.7%		
Employment status					
Employed	39 (26)	19.7%	33.9%		
Unemployed	109 (73)	65.4%	79.7%		
Refused to answer	1 (1)	.1%	4.7%		
Drugs injected, last 6 months^a^					
Buprenorphine	88 (59)	50.9%	66.7%		
Heroin	124 (83)	76.3%	88.5%		
Opium	15 (10)	6.1%	16.1%		
Methadone	25 (17)	11.5%	23.7%		
Other opioids	10 (7)	3.6%	12.1%		
Home-made stimulants	41 (28)	20.9%	35.3%		
Amphetamine/methamphetamine	30 (20)	14.4%	27.4%		
Cocaine/crack	4 (3)	1.0%	7.0%		
Antihistamines	1 (1)	.1%	4.7%		
Bio-amphetamine/bath salts	3 (2)	.6%	6.1%		
MDMA	1 (1)	.1%	4.7%		
Unknown substance	3 (2)	.6%	6.1%		
Did you share injection equipment with others, last month					
Yes	23 (15)	10.4%	22.2%		
No	125 (84)	77.0%	89.0%		
Do not know/do not remember	1 (1)	.1%	4.7%		
Age of first injection use		18.49	19.91	19.2 (4.4)	18 (13–37)
Years of regular injection use		12.89	16.08	14.5 (9.9)	14.0 (0.3–40)
Number of injections, last month		12.33	16.10	14.2 (11.7)	10 (1–60)
Total number of people drugs used with, last month^b^		4.45	6.49	5.5 (6.3)	3 (0–40)
Number of people injection instruments shared with, last month		2.79	4.17	3.5 (1.6)	3 (1–7)
Overdose experience, lifetime, any drug	83 (56)	47.6%	63.6%		
Number of overdose episodes, lifetime		2.27	4.92	3.6 (6.0)	2 (1–50)
Where did you get syringes from, last month					
Pharmacy only	127 (85)	78.5%	90.1%		
Friends only	4 (3)	1.0%	7.0%		
Pharmacy and friends	18 (12)	7.7%	18.4%		
Ever used NSP services					
Never	126 (85)	77.8%	89.6%		
Used earlier, more than 6 months ago	23 (15)	10.4%	22.2%		

^a^Sum exceeds 100% due to multi-drug use

^b^Drug use in a group, does not necessarily implies sharing of equipment

Heroin (83%), buprenorphine (58%), and amphetamine-type stimulants (including home-made preparations)(48%) were the most frequently injected drugs during the 6 months prior to the interview, and the vast majority (80%) reported using two or more substances during that period. Participants reported a mean of 14.2 (SD 11.7) injections per month (min = 1; max = 60). Group injection was prevalent and participants reported injecting with more than 5 different individuals (mean 5.5; SD 6.3) during the last 30 days. When asked to list their main sources for needles and syringes, 127 (85%) indicated pharmacies and 4 (3%) indicated friends, and 18 (12%) named both, pharmacy and friends, as a major source. Respondents reported no other sources from which they received/obtained needles and syringes in the last month. Fifteen percent of respondents reported sharing injection paraphernalia in the last 30 days, on average with 3.5 (SD 1.6) other people. The majority (*n* = 83; 56%) reported experiencing at least one (any drug-related) overdose episode in their life, and 11 (7%) reported having overdosed in the month prior to the interview. Three quarters of overdose cases were related to heroin injection. A significant majority (*n* = 126; 85%) reported never using NSP services, with more than half of the entire sample (*n* = 77; 52%) stating they never knew about these programs, and about a quarter of the sample (*n* = 36; 24%) believing they never needed these services. About a third of respondents (*n* = 45; 30%) said they have never been tested for HIV and the same proportion stated that they were tested for HIV (and know the results) during the last 12 months. Eighteen percent (*n* = 27) reported never being tested for HCV and 44% (*n* = 66) claimed they were tested for HCV (and know the results) during the last year.

### Perceived acceptability of syringe vending machines

All but one participant agreed that PWID would benefit from SVM and 145 (97%) said they would personally use SVM. When asked about the major reasons why they would use SVM, those who were willing to use machines most often named free of charge needle/syringes and other supplies (*n* = 61; 42%), no need to deal with pharmacies (*n* = 61; 42%), 24/7 working regime (*n* = 28; 19%), no need to wait in a line at a pharmacy counter (*n* = 15; 10%), and availability of HIV self-test kits (*n* = 14; 10%). Out of four respondents who indicated they would not utilize SVM, three explained that it was due to stigma (do not want to be seen using “drug addicts’ machine”) and one said he did not need it (see Table 2 for details). An overwhelming majority (*n* = 134; 90%) stated that they would use HIV self-tests if they were available from vending machines. Importantly, 84% (*n* = 38) of the 45 respondents who have never been tested for HIV said they would do testing if HIV self-tests were dispensed by SVM.

**Table 2:** Perceived acceptability of and willingness to use syringe vending machines (*N* = 149)

Variable	*N* (%)	95% CI	
		Lower	Upper
Do you believe SVMs will be beneficial for PWID?			
Yes	148 (99)	95.3%	99.9%
No	1 (1)	.1%	4.7%
Will you personally use SVM?			
Yes	145 (97)	93.0%	99.0%
No	4 (3)	1.0%	7.0%
Reasons for using SVM^a^			
No need to interact with pharmacy	61 (41)	33.3%	49.1%
Free of charge injection instruments	61 (41)	33.3%	49.1%
Accessible 24/7	28 (19)	13.2%	26.0%
Guaranteed confidentiality	15 (10)	6.1%	16.1%
Quick service, no need to wait in line (in pharmacy)	15 (10)	6.1%	16.1%
HIV and HCV self-tests	14 (9)	5.6%	15.3%
If injection instruments will be of high quality	7 (5)	2.2%	9.6%
If SVM will be conveniently located (in close proximity to me)	5 (3)	1.4%	7.9%
Contains all you need for injection	4 (3)	1.0%	7.0%
Because it will distribute naloxone	2 (1)	.3%	5.3%
You cannot buy some items in pharmacies due to fear of police	2 (1)	.3%	5.3%
Impossible to get everything you need in the pharmacy	1 (1)	.1%	4.7%
No need to interact with fixed NSP	1 (1)	.1%	4.7%
Do not know	1 (1)	.1%	4.7%
Will you perform HIV testing given self-test kits available from SVM?			
Yes	134 (90)	83.9%	93.9%
No	11 (7)	4.1%	12.9%
Prefer to do on-site testing	0 (0)	0%	0%
Not interested in testing at all	4 (3)	1.0%	7.0%
Will provision of general health consumables make SVMs more acceptable for general public?			
Definitely Yes	108 (72)	64.7%	79.1%
Probably Yes	32 (21)	15.6%	28.9%
Probably No	6 (4)	1.8%	8.7%
Definitely No	1 (1)	.1%	4.7%
Refuse to answer	0 (0)	0%	0%
Do not know	2 (1)	.3%	5.3%
Should SVM distribute kits for money?			
No	55 (37)	29.5%	45.0%
Yes, for less than it would cost to buy from pharmacy	75 (50)	42.3%	58.4%
Yes, for the same price as it would cost in pharmacy	16 (11)	6.6%	16.9%
Yes, for more than it would cost to buy from pharmacy	1 (1)	.1%	4.7%
Refuse to answer	0 (0)	0%	0%
Do not know	2 (1)	.3%	5.3%
Which of the following SVM access means do you prefer?			
Money	17 (11)	7.2%	17.7%
Multi-use permanent plastic card	117 (79)	71.1%	84.4%
Single-use coupon	14 (9)	5.6%	15.3%
Refuse to answer	1 (1)	.1%	4.7%

^a^Sum exceeds 100% due to multiple response options

### Items to be distributed from SVM

The questionnaire included a predefined list of health care products that could be vended by SVM, such as naloxone, pregnancy tests, and condoms. In addition, participants had an option to add other (not included in the list) items to the wish list. Table 3 presents the results and frequencies of respondent acceptability of these items. Standard items (such as needles and syringes, cotton and sterile water) received the highest endorsement. Notably, the majority (*n* = 83, 56%) supported inclusion of oral HIV self-test kits and almost half (*n* = 69, 46%) agreed that SVMs should distribute naloxone ampules as well.

**Table 3:** Injection equipment, other health products, and related items to be distributed via vending machines—participants’ wish list (*N* = 149)

Item	*N* (%)	95% CI	
		Lower	Upper
Needles and syringes	148 (99)	95.3%	99.9%
Cotton	89 (60)	51.6%	67.4%
Sterile water	88 (59)	50.9%	66.7%
Oral HIV self-test kit	83 (56)	47.6%	63.6%
Alcohol swab	82 (55)	46.9%	62.9%
Naloxone	69 (46)	38.4%	54.4%
Small glass vial	50 (34)	26.4%	41.6%
HCV self-test kit (if becomes available)	49 (33)	25.7%	40.9%
Male condom	47 (32)	24.5%	39.5%
Tourniquet	44 (30)	22.7%	37.4%
Scalp vein (butterfly) set	37 (25)	18.5%	32.5%
Contact information of available services	30 (20)	14.4%	27.4%
Risk reduction information and education materials	25 (17)	11.5%	23.7%
Filters	22 (15)	9.9%	21.5%
Cooking spoon	20 (13)	8.8%	20.0%
Female condom	18 (12)	7.7%	18.4%
Lemon acid^1^	16 (11)	6.6%	16.9%
Roller-bandage*	16 (11)	6.6%	16.9%
Medical plaster*	10 (7)	3.6%	12.1%
Pregnancy test kit	12 (8)	4.6%	13.7%
Iodine*	8 (5)	2.7%	10.4%
Hand napkins (wet)*	7 (5)	2.2%	9.6%
Antihistamine medicine*^,2^	7 (5)	2.2%	9.6%
Hand napkins (dry)*	6 (4)	1.8%	8.7%
Drug testing kit*	5 (3)	1.4%	7.9%
Surgical spirit*	4 (3)	1.0%	7.0%
Pain killer pills*	3 (2)	.6%	6.1%
Solid fuel tablets*^,3^	2 (1)	.3%	5.3%
Other*^,4^	7	–	–

*Items were not in a predefined list, but were added/proposed by participants

^1^To be added to low quality heroin

^2^Often mixed with opioids to increase the potency and prolong the effect

^3^Used to hit up heroin solution; also used at the final stage when preparing home-made stimulants or opioids to hit up drug solution; candle often used for similar purposes

^4^Items that received lowest endorsement (named only once) such as lighter, candle, urine testing kit and so on

The vast majority (*n* = 140; 93%) agreed that adding injection supplies and other hygienic/health products that would be utilized by the general population (for example condoms, hygienic tampons, nicotine patches) and would be available for purchase at a reasonable price from SVM could increase the acceptance of vending machines by local communities and neighborhoods.

### SVM access means and location

When asked about their preferences regarding the possible means to access SVM, 79% claimed the preference for a multi-use permanent plastic card (to be received at a fixed NSP site and to allow free access to SVM products), 11% stated they would prefer paying a reasonable price at the SVM (to avoid having to get in contact with a fixed harm reduction site to get access), and 9% stated they would prefer receiving single-use coupons for free products from their friends/peers who are in contact with fixed NSP.

The questionnaire included a list of possible specific locations to install SVMs (such as metro or bus stations, near pharmacies or markets), as well as a few descriptive characteristics about the locations to be considered (for example central streets, parks and others). Participants could also suggest new characteristics of preferred locations that were not in the predefined list. There was a diversity of opinions regarding possible locations for SVM. Locating SVM near metro stations received the highest endorsement (38%) and was followed by “near a residential building” (11%), “near a pharmacy”(10%), and “near a supermarket”(10%). Fifteen percent stated that SVMs should be installed on the sidewalks of central streets, but 11% voted for quiet, non-central, and non-crowded locations.

Because the overwhelming majority of respondents reported willingness to accept and use syringe vending machines, we did not perform a bivariate regression analysis for variables in the “SVM acceptability” domain as we originally had planned. Finally, there were no meaningful differences between weighted estimates and the sample raw estimates.

## Discussion

This study assessed the perceived acceptability of and willingness to use SVM among PWID in Tbilisi who were not in contact with harm reduction or substance use-related treatment services. The perceived acceptability of the novel syringe delivery model was extremely high. The most endorsed reasons provided by participants for potentially using SVM were free access to injection equipment and confidentiality. Other reasons included having uninterrupted access to injection instruments, fast service (no waiting in a queue in a pharmacy), and access to HIV self-testing kits.

The majority of participants in the sample were at very high risk for HIV, being poly-drug users, injecting in groups, and reporting recent sharing of injection instruments with others. The majority (85%) reported never having been in contact with a harm reduction program. In fact, the prevalence of injection risk behavior in this sample was notably higher than the prevalence reported by other authors in Georgian NSP clients and non-clients [7, 39], potentially due to their lack of contact with harm reduction or treatment facilities and low knowledge of harm reduction practices. Available studies have suggested that SVMs have the capacity to attract hard-to-reach and high-risk PWIDs [26, 30] and those who have never been in substance use-related treatment [40]. If SVMs in Georgia are able to attract PWID like those in this study sample, they could, at a minimum, substantially expand access to sterile equipment for wider groups of very high-risk drug users, but also could facilitate linkage to prevention and treatment services, offering substantial public health benefits. Like NSP, SVM could facilitate the linkage with substance use-related and general health services [15, 41]. Importantly, almost 80% of those who confirmed they would be willing to use SVM claimed that they would prefer to receive a multi-use plastic card to have permanent access to machines. This suggests that they might be willing to get in contact with a fixed NSP through which SVM plastic cards would be distributed.

Eight out of ten participants who have never been tested for HIV stated that they would take an HIV test if self-test kits were available through vending machines. A number of countries have already started implementing HIV self-testing or included it in their strategic plans or policy frameworks [44]. In Georgia the significant gap in HIV case detection (only 48% of the estimated number of people living with HIV is aware of their status) [45] prompted the government to acknowledge the need for engaging out-of-service PWID (testing, early diagnosis, inclusion in treatment) as a critical priority for the HIV national response. SVMs and HIV self-testing are both included in the HIV/AIDS National Strategic Plan 2019–2022 as innovative and potentially beneficial approaches for the country [45]. There is a growing volume of research assessing the feasibility, acceptability, and accuracy of HIV self-tests. This research has suggested that self-testing is a reliable and convenient form of testing for many individuals and groups who may not test otherwise, including key affected populations [46–49]. Self-testing is highly acceptable among various users in different settings [44, 50–55] and increases access to and the uptake and frequency of HIV testing [56–58]. In addition, feasibility and acceptability studies suggest that providing HIV self-testing kits through vending machines has the potential to improve testing rates in key risk groups [42, 43]. However, there are concerns regarding the further confirmatory HIV testing and linkage to care [44, 59]. We certainly acknowledge that there is a need for research, including in Georgia, to assess strategies that would ensure that self-testing leads to more people knowing their HIV status and more HIV-positive individuals linked to treatment and care. There has been promising research showing that self-testing could facilitate HIV case-finding in some settings [60] and post-test linkage to care using community-based proactive interventions [61].

As expected, the majority of participants were willing to receive from vending machines a relatively standard set of items used for drug preparation and injection—needles and syringes, cotton, sterile water, and alcohol swabs. There also were high rates of endorsement for HIV and HCV (if/when available) self-test kits, as well as for naloxone. In addition, participants indicated moderate endorsement of female condoms and pregnancy kits and proposed to include some new (not in a predefined list) items, such as drug-testing kits. These results suggest potential broader utility of vending machines and the need to experiment with distribution of a wider variety of consumables and health products beyond the relatively narrow, traditional set of items that are usually vended from SVMs.

As expected, the vast majority of our sample had obtained needles and syringes in pharmacies. This finding is similar to findings from other PWID-focused studies in Georgia [22]. Needles, syringes, and other injection paraphernalia are available via unrestricted sale through pharmacies, some of which provide 24/7 service to customers. However, strict drug-related legislation, police harassment, and severe social stigma associated with drug use represent major barriers to PWID obtaining syringes in pharmacies (and, in some cases, to using prevention and treatment services as well) [9]. In many cases, female partners of drug users are tasked to show up at pharmacies to buy injection supplies for their drug-using male partners [62, 63]. Not surprisingly, the overwhelming majority of respondents in this study ranked confidentiality (no need to deal with pharmacies), as a principal advantage to using SVM. Similar to our findings, a number of other authors have found confidentiality and stigma-free access to sterile equipment to be important factors in attracting PWID to syringe distribution modalities [26, 30].

We had only one female and a very few young (18–25 years of age) respondents in the sample. Although we invited a female seed as initial recruiter to the study and hoped she would recruit other female PWID, she did not recruit any participants. The lack of drug-using women and young people in contact with substance use-related services and their extremely low representation in PWID-focused studies reflect a common trend in Georgian drug-related service delivery and research setting. Females and young drug users represent a tiny fraction (1–5%) of clients in harm reduction and substance use-related treatment services in the country [8, 64]. Previous research has shown that social and cultural stigmatization and lack of services shape the help-seeking behavior of these populations [65, 66]. Research in other settings suggests that compared to traditional NSP, SVMs are more likely to attract younger PWID, female PWID, as well as others who are not likely to be in treatment [27, 28, 30]. Given this potential for attracting hard-to-reach sub-groups of PWID, implementation of SVM in Georgia could offer a breakthrough to harm reduction efforts across the country.

Given the issues of stigmatization and need for confidentiality in Georgia and other legally and socio-culturally constrained settings, SVM implementation needs to address a number of environmental challenges and employ risk minimization strategies. There was little agreement among respondents on the locations for installing SVMs. Interestingly, major concerns were related to possible resistance from residents and the general public, and there was less fear regarding police harassment since many believed that “police know all drug users anyway”. Most arguments for or against any specific locations were driven by participants’ willingness to make the process either less visible for outsiders (quiet, distant locations) or to employ approaches that would reduce the possibility of labelling the SVM as a vending machine for PWID. One such approach to mitigating stigma was our suggestion to have SVMs distributing simple health products for the general population. This suggestion received approval from the vast majority of respondents in our sample. We acknowledge that at this stage, it is difficult to propose any strategy that would assure acceptance of SVMs by the general public or different groups of PWID. Having SVMs distributing general health products is just one possible means to counter potential risks. Further research will illuminate more about these and other challenges as we implement the first two SVMs, planned within our ongoing SVM trial.

### Limitations

The sample was small and not randomly selected, participants were almost exclusively men, and we had very low representation of young PWID. This limits our ability to apply our findings to the entire out-of-service PWID population in Tbilisi. General concern related to recall bias might be applicable to the current study as well. The authors do not exclude the possibility of social desirability bias while reporting needle and syringe sharing. However, we believe that its effect, if any, was minimal in this sample. Respondents were service-naïve, interviewers were unknown to them, and there were fewer expectations of being in future contact with interviewers. In addition, it is unlikely that respondents had common understanding of what might be considered as a “correct” or “good” response to the question of sharing. Notably, the prevalence of reported sharing in our sample was the highest prevalence of sharing across any recent published reports for Georgia, and thus, the number does not seem to be underreported.

Results of the weighted analysis did not differ meaningfully from the raw estimates. RDS weights correct for differences in respondent network size (also referred to as “degree”) and transition probabilities across groups (i.e., the probability that a person will differentially recruit from groups with characteristics different from one’s own, during recruitment, allowing for unbiased population prevalence estimates) [67]. The goal of the RDS method is the same as that of other probability sampling methods: to make adjustments to raw estimates derived from the recruited sample that account for sampling bias (i.e., each member of the group having a different probability of being included in the sample) in order to make inferences about the population from which the sample was drawn [67]. The primary purpose of employing RDS was to reach hidden groups of PWID who were not utilizing services. Characteristics of the study participants and patterns of drug use-related behaviors suggest that we accomplished this goal; participants were primarily very high-risk PWID who had never been in contact with any services. Limitations notwithstanding, characteristics of the sample and attitudes about preferences for SVM provide valuable input into the principal questions posed by the study.

Of note, following the completion of interviews, 28 respondents from our sample did first-ever HIV/HCV testing at the site; 11 were HCV+, and 8 were linked to and started on HCV treatment with direct-acting antivirals. (Note: biological testing was not part of the study and was offered routinely to study participants as a standard care at the NSP site that hosted the fieldwork for the survey.)

## Conclusions

Acceptability of SVM was extremely high, with the most highly endorsed features being uninterrupted free access to sterile injection equipment and privacy and anonymity. In addition, acceptability of self-testing HIV kits was also high, including among PWID who have never been tested for HIV. Results of this study suggest that introducing SVM in Georgia holds the potential to deliver significant public health benefits through attracting hard-to-reach PWID, reducing unsafe injection behavior, and facilitating HIV testing and linkage to care.

## Abbreviations

EECA: Eastern Europe and Central Asia
GEL: Georgian Lari
GFATM: Global Fund to Fight AIDS, Tuberculosis and Malaria
GHRN: Georgian Harm Reduction Network
HCV: Hepatitis C virus
HIV: Human immunodeficiency virus
NSP: Needle and syringe program
OST: Opiate substitution program
PWID: People who inject drugs
RDS: Respondent-driven sampling
SVM: Syringe vending machine
UNAIDS: Joint United Nations Program on HIV/AIDS
USD: United States Dollar

## References

[1] Harm Reduction International. The Global State of Harm Reduction 2016. London: Harm Reduction International; 2016.

[2] UNODC. World Drug Report 2017 Vienna, Austria: UNODC 2017.

[3] Bemoni Public Union, Curatio International Foundation. Estimating the prevalence of injection drug use in Georgia. Tbilisi, Georgia: Bemoni Public Union 2016.

[4] Fettig J, Swaminathan M, Murrill CS, Kaplan JE (2014). Global epidemiology of HIV. Infect Dis Clin N Am.

[5] DeHovitz J, Uuskula A, El-Bassel N (2014). The HIV epidemic in Eastern Europe and Central Asia. Curr HIV/AIDS Rep.

[6] UNAIDS. Ending AIDS: progress towards the 90–90–90 targets: UNAIDS2017.

[7] Curatio International Foundation, Public Union Bemoni. HIV risk and prevention behaviours among people who inject drugs in six cities of Georgia: bio-behavioral surveillance survey in Tbilisi, Batumi, Zugdidi, Telavi, Gori, Kutaisi in 2014. Tbilisi 2015.

[8] Alavidze S, Duchidze N, Kirtadze I, Otiashvili D, Razmadze M., Sturua L., et al. The drug situation in Georgia, Annual Report 2015. Tbilisi 2016.

[9] Otiashvili D, Tabatadze M, Balanchivadze N, Kirtadze I (2016). Policing, massive street drug testing and poly-substance use chaos in Georgia–a policy case study. Subst Abuse Treat Prev Policy.

[10] Georgia Infectious Diseases AIDS and Clinical Immunology Research Center. HIV/AIDS epidemiology in Georgia. 2016. Available from: http://aidscenter.ge/epidsituation_eng.html. Accessed 24 Nov 2016.

[11] UNAIDS (2016). Prevention gap report. July 11, 2016.

[12] ECDC (2015). HIV/AIDS surveillance in Europe: European Centre for Disease Prevention and Control.

[13] Aspinall EJ, Nambiar D, Goldberg DJ, Hickman M, Weir A, Van Velzen E (2014). Are needle and syringe programmes associated with a reduction in HIV transmission among people who inject drugs: a systematic review and meta-analysis. Int J Epidemiol.

[14] Abdul-Quader AS, Feelemyer J, Modi S, Stein ES, Briceno A, Semaan S (2013). Effectiveness of structural-level needle/syringe programs to reduce HCV and HIV infection among people who inject drugs: a systematic review. AIDS Behav.

[15] WHO (2014). Consolidated guidelines on HIV prevention, diagnosis, treatment and care for key populations.

[16] Wilson D, Zhang L, Kerr C, Kwon A, Hoare A, Otiashvili D (2012). Evaluating the cost-effectiveness of needle-syringe exchange programs in Georgia.

[17] Wilson DP, Donald B, Shattock AJ, Wilson D, Fraser-Hurt N (2014). The cost-effectiveness of harm reduction. Int J Drug Policy.

[18] WHO, UNODC, UNAIDS. Technical guide for countries to set targets for universal access to HIV prevention, treatment and care for injecting drug users. Geneva: World Health Organization; 2012.

[19] Otiashvili D (2015). Situation analysis of sustainability planning and readiness for responsible transition of harm reduction programs from Global Fund support to national funding in EECA Vilnius.

[20] Georgian Harm Reduction Network (2017). GHRN program database.

[21] Beselia A, Gegenava V, Kirtadze I., Mgebrishvili T, Otiashvili D, Razmadze M., et al. Drug situation in Georgia 2016–2017. Tbilisi, Georgia: Alternative Georgia; 2018.

[22] Curatio International Foundation & Bemoni Public Union. HIV risk and prevention behaviors among people who inject drugs in seven cities of Georgia. Tbilisi, Georgia: Curatio International Foundation; 2017.

[23] Kirtadze I. Harm reduction program cost optimisation assessment. Tbilisi, Georgia: Georgian Harm Reduction Network 2018.

[24] Simionov V. Count the costs, Romania country report 2013: Bucharest, Romanian Harm Reduction Network; 2013.

[25] Furtunescu F. Sustainability of Global Fund supported programs: what happened before and after the Global Fund left? Bucharest: Romanian Harm Reduction Network; 2015.

[26] Islam MM, Conigrave KM (2007). Assessing the role of syringe dispensing machines and mobile van outlets in reaching hard-to-reach and high-risk groups of injecting drug users (IDUs): a review. Harm Reduct J.

[27] Islam MM, Conigrave KM (2007). Syringe vending machines as a form of needle syringe programme: advantages and disadvantages. J Substance Use.

[28] Moatti JP, Vlahov D, Feroni I, Perrin V, Obadia Y (2001). Multiple access to sterile syringes for injection drug users: vending machines, needle exchange programs and legal pharmacy sales in Marseille. France Eur Addict Res.

[29] Cama E, Brener L, Bryant J. Characteristics and attendance patterns of a fixed-site NSP and nearby SVM: The benefits of 24-hour access to sterile injecting equipment. Drugs, 2014;21(6):476–81.

[30] Jones L, Pickering L, Sumnall H, McVeigh J, Bellis MA (2010). Optimal provision of needle and syringe programmes for injecting drug users: a systematic review. Int J Drug Policy..

[31] McDonald D. ACT syringe vending machines trial 2005–2006: Siggins Miller 2007.

[32] Government of Western Australia. Metropolitan needle and syringe vending machine trial: evaluation report: Department of Health, Sexual Health and Blood-borne Virus Program 2014.

[33] Abdul-Quader AS, Heckathorn DD, McKnight C, Bramson H, Nemeth C, Sabin K (2006). Effectiveness of respondent-driven sampling for recruiting drug users in New York City: findings from a pilot study. J Urban Health.

[34] Heckathorn D (2002). Respondent driven sampling II: deriving valid population estimates from chain-referral samples of hidden populations. Soc Probl.

[35] Heckathorn D (1997). Respondent driven sampling: a new approach to the study of hidden samples. Soc Probl.

[36] McKnight C, Des Jarlais D, Bramson H, Tower L, Abdul-Quader AS, Nemeth C (2006). Respondent-driven sampling in a study of drug users in New York City: notes from the field. J Urban Health.

[37] Heckathorn DD (2011). Snowball versus respondent-driven sampling. Sociol Methodol.

[38] Public Union Bemoni & Curatio International Foundation. Population size estimation of people who inject drugs in Georgia 2016. Tbilisi, Georgia: Bemoni Public Union; 2017.

[39] Gogia M. Assessing risky behavior of PWIDs in Georgia - comparable analyses of two samples. J Human Virol Retrovirol. 2015;2(3).

[40] Obadia Y, Feroni I, Perrin V, Vlahov D, Moatti JP (1999). Syringe vending machines for injection drug users: an experiment in Marseille, France. Am J Public Health.

[41] Wodak A, Cooney A (2004). Effectiveness of sterile needle and syringe programming in reducing HIV/AIDS among injecting drug users.

[42] Young SD, Daniels J, Chiu CJ, Bolan RK, Flynn RP, Kwok J (2014). Acceptability of using electronic vending machines to deliver oral rapid HIV self-testing kits: a qualitative study. PLoS One.

[43] Young S, Klausner J, Fynn R, Bolan R (2014). Electronic vending machines for dispensing rapid HIV selftesting kits: a case study. AIDS Care.

[44] Figueroa C, Johnson C, Verster A, Baggaley R (2015). Attitudes and acceptability on HIV self-testing among key populations: a literature review. AIDS Behav.

[45] Government of Georgia. Georgia HIV/AIDS National Strategic Plan 2019–2022. Tbilisi, Georgia: Ministry of Labour Health and Social Affairs of Georgia; 2018.

[46] WHO. Consolidated guidelines on HIV testing services. Geneva, Switzerland: World Health Organization; 2016 [cited 2018 19 August]; Available from: http://www.who.int/hiv/pub/guidelines/hiv-testing-services/en/.

[47] WHO. Guidelines on HIV self-testing and partner notification: supplement to consolidated guidelines on HIV testing services. Geneva, Switzerland: World Health Organization 2016. 27977094

[48] WHO/UNAIDS. A short technical update on self-testing for HIV. Geneva, Switzerland: World Health Organization; 2014.

[49] Johnson C, Baggaley R, Forsythe S, van Rooyen H, Ford N, Napierala Mavedzenge S (2014). Realizing the Potential for HIV self-testing. AIDS Behav.

[50] Martin IB, Williams V, Ferguson D, Read S. Performance of and preference for oral rapid HIV testing in the Bahamas. Journal of Infection and Public Health. 2017 2017/07/03/. 10.1016/j.jiph.2017.06.005 28684223

[51] Pant Pai N, Joshi R, Dogra S, Taksande B, Kalantri SP, Pai M (2007). Evaluation of diagnostic accuracy, feasibility and client preference for rapid oral fluid-based diagnosis of HIV infection in rural India. PLoS One.

[52] Marley G, Kang D, Wilson EC, Huang T, Qian Y, Li X (2014). Introducing rapid oral–fluid HIV testing among high risk populations in Shandong, China: feasibility and challenges. BMC Public Health.

[53] Lee VJ, Tan SC, Earnest A, Seong PS, Tan HH, Leo YS (2007). User acceptability and feasibility of self-testing with HIV rapid tests. J Acquir Immune Defic Syndr.

[54] Ng OT, Chow AL, Lee VJ, Chen MIC, Win MK, Tan HH (2012). Accuracy and user-acceptability of HIV self-testing using an oral fluid-based HIV rapid test. PLoS One.

[55] Carballo-Dieguez A, Frasca T, Balan I, Ibitoye M, Dolezal C (2012). Use of a rapid HIV home test prevents HIV exposure in a high risk sample of men who have sex with men. AIDS Behav.

[56] Choko AT, MacPherson P, Webb EL, Willey BA, Feasy H, Sambakunsi R (2015). Uptake, accuracy, safety, and linkage into care over two years of promoting annual self-testing for HIV in Blantyre, Malawi: a community-based prospective study. PLoS Med.

[57] Tao J, Li MY, Qian HZ, Wang LJ, Zhang Z, Ding HF (2014). Home-based HIV testing for men who have sex with men in China: a novel community-based partnership to complement government programs. PLoS One.

[58] Bavinton BR, Brown G, Hurley M, Bradley J, Keen P, Conway DP (2013). Which gay men would increase their frequency of HIV testing with home self-testing?. AIDS Behav.

[59] Pant Pai N, Sharma J, Shivkumar S, Pillay S, Vadnais C, Joseph L, et al. Supervised and unsupervised self-testing for HIV in high- and low-risk populations: a systematic review. PLoS Medicine. 2013 04/02 07/20/received 02/22/accepted;10(4):e1001414. 10.1371/journal.pmed.1001414 PMC3614510 23565066

[60] Kurth AE, Cleland CM, Chhun N, Sidle JE, Were E, Naanyu V (2016). Accuracy and acceptability of oral fluid HIV self-testing in a general adult population in Kenya. AIDS Behav.

[61] MacPherson P, Lalloo DG, Webb EL (2014). Effect of optional home initiation of HIV care following HIV self-testing on antiretroviral therapy initiation among adults in Malawi: a randomized clinical trial. JAMA..

[62] Kirtadze I, Otiashvili D, O’Grady K, Zule W, Krupitsky E, Wechsberg W (2015). Women who inject drugs in the Republic of Georgia: in their own words. J Psychoactive Drugs.

[63] Lund I, Kirtadze I, Otiashvili D, O’Grady K, Jones H (2012). Female partners of opioid-injecting men in the Republic of Georgia: an initial characterization. Subst Abuse Treat Prev and Policy.

[64] Otiashvili D, Kirtadze I, O’Grady KE, Zule W, Krupitsky E, Wechsberg WM (2014). Comprehensive women-centered treatment for substance use disorders in Georgia: current status and future directions. J Subst Abus.

[65] Otiashvili D, Kirtadze I, O’Grady KE, Zule W, Krupitsky E, Wechsberg WM (2013). Access to treatment for substance-using women in the Republic of Georgia: socio-cultural and structural barriers. Int J Drug Policy.

[66] Kirtadze I, Otiashvili D, O’Grady KE, Zule WA, Krupitskii EM, Wechsberg WM (2013). Twice stigmatized: health service provider’s perspectives on drug-using women in the Republic of Georgia. J Psychoactive Drugs.

[67] Heckathorn D (2007). Extensions of respondent-driven sampling: analyzing continuous variables and controlling for differential recruitment. Sociol Methodol.

